# Novel Insights Into Immune Systems of Bats

**DOI:** 10.3389/fimmu.2020.00026

**Published:** 2020-01-24

**Authors:** Arinjay Banerjee, Michelle L. Baker, Kirsten Kulcsar, Vikram Misra, Raina Plowright, Karen Mossman

**Affiliations:** ^1^Department of Pathology and Molecular Medicine, Michael DeGroote Institute for Infectious Disease Research, McMaster Immunology Research Centre, McMaster University, Hamilton, ON, Canada; ^2^Health and Biosecurity Business Unit, Australian Animal Health Laboratory, CSIRO, Geelong, VIC, Australia; ^3^Department of Microbiology and Immunology, University of Maryland School of Medicine, Baltimore, MD, United States; ^4^Department of Veterinary Microbiology, Western College of Veterinary Medicine, University of Saskatchewan, Saskatoon, SK, Canada; ^5^Department of Microbiology and Immunology, Montana State University, Bozeman, MT, United States

**Keywords:** bats (Chiroptera), virus, innate and adaptive immune response, interferon, antiviral, emerging viruses

## Abstract

In recent years, viruses similar to those that cause serious disease in humans and other mammals have been detected in apparently healthy bats. These include filoviruses, paramyxoviruses, and coronaviruses that cause severe diseases such as Ebola virus disease, Marburg haemorrhagic fever and severe acute respiratory syndrome (SARS) in humans. The evolution of flight in bats seem to have selected for a unique set of antiviral immune responses that control virus propagation, while limiting self-damaging inflammatory responses. Here, we summarize our current understanding of antiviral immune responses in bats and discuss their ability to co-exist with emerging viruses that cause serious disease in other mammals. We highlight how this knowledge may help us to predict viral spillovers into new hosts and discuss future directions for the field.

## Introduction

The outbreaks of Nipah virus infection in Malaysia (1998–1999) and Bangladesh (2001), Hendra virus infection in Australia (1994) and the severe acute respiratory syndrome (SARS) pandemic of 2003 laid the foundations for investigations into bats (mammals of the order Chiroptera) as reservoirs of emerging viruses (1, 2). During the SARS pandemic of 2003, 8,098 people were infected and 774 died (3). Subsequent sampling identified a closely related SARS-like virus in Himalayan palm civets (*Paguma larvata*) (4) and bats in the region (2, 5). Moreover, viruses similar to those that cause serious diseases in humans and agricultural animals have been detected in bats. These include filoviruses (related to Ebola and Marburg viruses) (6–8), paramyxoviruses (related to Nipah and Hendra viruses) (9) and coronaviruses [related to viruses that cause SARS and Middle East respiratory syndrome (MERS)] (10–14). Of note, these viruses do not appear to cause disease in bats and this has led to multiple studies exploring the ability of bats to harbor these viruses with no observed clinical consequence (15–17).

The order Chiroptera is diverse and consists of over 1300 species of bats that are distributed across every continent except Antarctica (18). Chiroptera consists of two suborders, Yinpterochiroptera (which comprises megabats and several families of microbats) and Yangochiroptera (comprising all remaining microbat families) that diverged over 50 million years ago (19–21). Within these suborders, bat species display tremendous diversity in size, morphology, ecological niches, diets, and social interactions. Considering the large number of species and the evolutionary diversification, studies performed on one bat may not be representative of all species. Bats play an important role in the ecosystem via pollination, seed dispersal and insect control (18). However, recent studies have also discovered an increasing diversity of zoonotic viruses in bats (22, 23). Experimental infections of multiple bat species with Ebolavirus (24), Marburg virus (MARV) (25), MERS coronavirus (MERS-CoV) (26), Nipah virus (27), and Hendra virus (28) have demonstrated limited viraemia in bats. These studies raise further questions about adaptations in bat antiviral immune responses. The evolution of the bat antiviral immune system is multifaceted and several factors, such as the evolution of flight (29, 30) and co-evolution of bats with their viruses have likely shaped their distinct immunological responses. Understanding how bats control virus-mediated pathogenesis may enable researchers to identify novel therapeutic targets and molecules to treat infections with these viruses in other mammals, including humans and agricultural animals. In this Review, we summarize major developments in understanding bat antiviral responses, highlighting unique properties of bat immune systems and comparing and contrasting antiviral signaling pathways in bat and human cells.

## Innate Immunity in Bats

Mammalian cells have evolved conserved pattern recognition receptors (PRRs) that sense pathogen associated molecular patterns (PAMPs) derived from viruses, bacteria and parasites (31–33). Following virus infection, infected cells initiate signaling events that induce the expression of antiviral and pro-inflammatory cytokines (33–35). Antiviral cytokines, such as interferons (IFNs), activate the expression of IFN-stimulated genes (ISGs) that inhibit virus replication through different mechanisms [reviewed by Schoggins et al. (36)]. Innate signaling pathways are being extensively investigated in human and rodent cells and recent studies have discovered the existence of similar pathways in bats.

The availability of whole genome and transcriptome sequences for some bat species has enabled *in silico* data mining to detect homologs of the mammalian innate immune system. Approximately 3.5% (or 500 in total) of the transcribed genes identified in the black flying fox (*Pteropus alecto*) are thought to be immune-related (37). In the closely related Jamaican fruit bat (*Artibeus jamaicensis*), 466 immune-related genes have been identified by transcriptome analysis (38) and 2.75% of genes (roughly 407 genes) in the Egyptian fruit bat (*Rousettus aegyptiacus*) are immune-related (39). These studies were amongst the first to produce transcriptomic datasets for bats and pioneered studies of the bat immune system. Furthermore, an examination of the transcriptome of *P. alecto* identified a proportion of transcripts that did not match known annotated transcripts, suggesting the presence of bat-specific transcripts, some of which may also have immune functions (37). In comparison, 7% of the human genome represents immune genes (40). Thus, it is possible that we are yet to discover the full range of immune related genes in bats or bats may indeed have a smaller repertoire of immune-related genes, relative to humans. These studies need to be further validated by an exhaustive search of novel immune-related genes in bats, sampling multiple bat species and sequencing transcripts from different cell types and tissues. The genomes and transcriptomes of at least 18 bat species are currently available in databases (30, 41), providing important insights into the evolution of their immune system and antiviral immunity. Below, we discuss the evolution of antiviral responses in bats in the context of cellular detection of RNA and DNA viruses.

### Bat PRRs and RNA Viruses

PRRs, such as Toll-like receptors (TLRs) are evolutionarily conserved across the animal kingdom (42). Due to the importance of bats as reservoirs of zoonotic RNA viruses (43), there is particular interest in identifying the intracellular PRRs in bat cells that may engage antiviral signaling pathways following infection with RNA viruses. In human cells, endosomal TLRs 3, 7, and 8 detect viral RNAs (35). Full-length transcripts for TLR 1- TLR10 have been sequenced in *P. alecto* and a TLR13 pseudogene has been detected (44), but their functions in bats have not been fully characterized. Bat cells from multiple species upregulate type I IFNs and ISGs in response to poly(I:C) treatment and Sendai virus infection, suggesting that the dsRNA sensing machinery is conserved between bat and human cells (45–49). The role of TLR3 in sensing exogenous dsRNA has been confirmed in cells from the big brown bat (*Eptesicus fuscus*) (49), but interaction studies and identification of ligand binding domains in bat TLR3 have not been performed. Computational structural analysis of *TLR8* sequences from twenty-one bat species has identified differences between bat and human sequences (50). 63% of bat *TLR8* genes have evolved under purifying selection and 7% of amino acid sequences that make up the ligand-binding domain of TLR8 differ between bat and other mammalian TLR8 proteins. Moreover, *TLR8* sequences vary within bat species (50). Thus, it is important to acknowledge species-specific adaptations in bats.

Cytosolic PRRs, such as retinoic acid-inducible gene-I (RIG-I) and melanoma differentiation-associated gene 5 (MDA5), that detect exogenous RNA in human cells have been detected in most bat genomes or transcriptomes that have been studied (37, 49). RIG-I and MDA5 from *P. alecto* have similar primary structures and patterns of tissue expression compared to their human counterparts. Similar to human and rodent cells, *P. alecto* kidney cells produce IFNs in response to stimulation with poly(I:C) (46). A separate study in the distantly related insectivorous bat species, *E. fuscus* also identified a role for RIG-I and MDA5 in sensing poly(I:C) (49). Thus, cytosolic RNA sensors are conserved and functional in bat cells.

### Limited Inflammatory Responses

**Figure 1:**
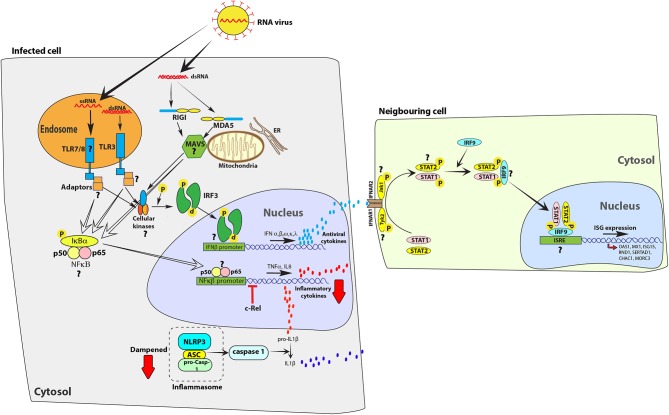
Bat cells mount an antiviral response to RNA viruses, but limit the expression of inflammatory cytokines. Infection with RNA viruses, such as Sendai virus, or transfecting cells with surrogate double-stranded RNA [poly(I:C)] or single-stranded RNA is detected by Toll-like receptors (TLRs) 3, 7 and 8 or cytosolic receptors retinoic acid-inducible gene-I (RIG-I) and melanoma differentiation-associated protein 5 (MDA5). Activation of these receptors activate downstream adaptor proteins, such as mitochondrial antiviral signaling protein (MAVS). Adaptor proteins activate cellular kinases, such as TANK-binding kinase 1 protein (TBK1), which in-turn activate interferon regulatory factor 3 (IRF3) or IRF7 and nuclear factor kappa-light-chain-enhancer of activated B cells (NFκB) to stimulate the expression of interferons (IFNs), such as IFNs α, β, ω, κ, and λ, and pro-inflammatory cytokines, such as IL8, TNFα, and IL1β, respectively. IFNs bind to interferon α/β receptors (IFNAR; IFNAR1 and IFNAR2) on infected and neighboring cells to activate the JAK-STAT signaling pathway via kinases such as Janus kinase 1 (Jak1) and tyrosine kinase 2 (Tyk2) that phosphorylate signal transducer and activator of transcription (STAT) proteins. Phosphorylated STAT proteins (STAT1 and STAT2) merge with IRF9 and induce the expression of interferon stimulated genes (ISGs), such as OAS1 and Mx1. However, unlike in human cells, the parallel activation of pro-inflammatory cytokines is dampened in bat cells. c-Rel, a protein from the NFκB family of proteins binds to the TNFα promoter to inhibit activation of this pro-inflammatory cytokine in *E. fuscus* cells (49). The bat NLRP3 inflammasome activation is dampened, reducing the ability of bat cells to produce IL1β, a key inflammatory cytokine (17). In the figure, red arrows indicate a dampened response in the pathway, relative to human cells. Question marks (**?**) highlight pathways and molecular homologs that have not been characterized or identified in bats. The data have been compiled from studies in different species and one finding may not represent a universal bat response. ER, endoplasmic reticulum.

Following ligand sensing, PRRs signal through adaptor proteins to express antiviral and pro-inflammatory cytokines. Regulation of such inflammatory responses is crucial in order to limit tissue damage. Many severe virus infections are associated with excessive inflammation-associated pathology in humans (51, 52). Bats have evolved novel mechanisms to limit virus-induced pro-inflammatory responses while maintaining type I IFN responses to limit virus propagation (Figure 1). Understanding how bats limit virus-induced pro-inflammatory processes may enable researchers to adapt these strategies to counteract inflammation in humans.

In *E. fuscus*, the NF-κB family member c-Rel can interact with the promoter sequences of *TNF* to restrict the levels of production of this inflammatory cytokine (49). The lack of a strong inflammatory response in virally infected bat immune cells has also been attributed to low levels of NLRP3 inflammasome activation (17). Bat cells infected with influenza A viruses, Melaka virus or MERS-CoV induced lower levels of apoptosis-associated speck-like protein containing a CARD (ASC) speck formation and IL-1β secretion compared to what is seen in mouse and human cells, but this had minimal effect on the levels of virus replication (17). Strong and chronic inflammation has been associated with poor disease prognosis and health issues in humans and other susceptible animal models (53, 54). The ability of bats to control high levels of inflammation may also explain their long life span (55, 56) in addition to their ability to host (57) multiple viruses in the absence of clinical disease. Similar studies on virus-host interactions in bats are allowing researchers to understand the evolution of antiviral responses in mammals and underlying factors that lead to lethal disease outcomes in humans upon infection with emerging bat-borne viruses.

### Induction of IFNs in Bats

In human cells, RNA and DNA virus recognition and signaling converge on the transcription factors interferon regulatory factor 3 (IRF3) and IRF7 (31, 58) that drive the expression of IFNs (33). Bat *IRF3* sequences are evolutionarily distinct from their mammalian counterparts (59). Functional studies indicate that IRF3 in *E. fuscus* cells mediates antiviral signaling in response to poly(I:C) and MERS-CoV. Knock-down or deficiency of *IRF3* in *E. fuscus* cells reduces IFNβ induction in response to polyI:C stimulation or MERS-CoV infection (59). In *P. alecto* cells, *IRF7* mRNA is constitutively expressed and has a more widespread tissue distribution in bats compared to in humans and mice, which may allow bats to respond more rapidly to infection (60). Functional activity of *P. alecto* IRF7 has also been demonstrated. Similarly to knockdown of *IRF3* mRNA in *E. fuscus*, knockdown of *IRF7* in *P. alecto* cells significantly reduced the induction of mRNA encoding IFNβ following infection with the mouse paramyxovirus Sendai virus and led to an increase in viral titres when the cells were infected with the bat paramyxovirus Pulau virus (60). In both *P. alecto* and *E. fuscus* cells, IRF7 is induced in response to poly(IC) stimulation (49, 60). However, cytoplasmic adaptor proteins of TLRs and cellular kinases that activate IRF3 and IRF7 have not been studied in bats.

The RLR adaptor, mitochondrial antiviral-signaling (MAVS) protein leads to nuclear translocation of NF-κB and IRF3 for the induction of type I IFNs (61). Functional conservation in MAVS signaling has been demonstrated in the Chinese rufous horseshoe bat (*Rhinolophus sinicus)* and the straw-colored fruit bat (*Eidolon helvum)*. Interestingly, expression of bat MAVS in MAVS knockout human cells resulted in induction of the IFNβ promoter and expression of an ISG, namely IFN-induced protein with tetratricopeptide repeats 1 (IFIT1) (62). Expression of bat and rodent MAVS in human MAVS knockout cells also resulted in the activation of IRF3 post Sendai virus infection (62). These studies suggest that rodent, human and bat MAVS have conserved functional properties, however, downstream signaling pathways and molecules involved in MAVS-mediated signaling are yet to be characterized in bats.

An early IFN response is critical to limit virus propagation (36, 63, 64). The type I IFN encoding locus is contracted in the *P. alecto* genome; it has only 10 *IFN* loci, including three functional *IFN*α loci, and this is fewer than any other mammalian species (15). Unstimulated tissues and cells from *P. alecto* constitutively express transcripts for three IFNα genes and associated ISGs. In contrast, constitutive expression of IFNα was not observed in primary cells from *R. aegyptiacus* (16), hinting at species-specific differences in IFN responses in bats. Treating *R. aegyptiacus* cells with IFNω functionally inhibited vesicular stomatitis virus (VSV) replication (16). Sendai virus infection also induces the expression of IFNs in *R. aegyptiacus* cells, including an IFNω response (16). IFNκ and IFNω from the Serotine bat (*Eptesicus serotinus*) can also limit replication of multiple lyssavirus strains in susceptible bat cell lines (65).

Beyond type I IFNs, type III IFNs (IFNλs) also play a role in antiviral immunity in mammals and induce a similar subset of ISGs (66). *P. vampyrus* has coding sequences for three type III IFNs in its genome, which is similar to the number of functional type III IFNs in humans, but only two are transcribed in the closely related bat *P. alecto* (67). Expression of IFNλ1 and λ2 in *P. alecto* splenocytes was induced upon infection with Tioman virus (bat paramyxovirus) in the absence of type I IFN expression, providing evidence for a role for type III IFNs in the ability of bats to coexist with viruses (67). However, henipavirus infection antagonizes type I and type III IFN production and signaling in *P. alecto* cells, unlike in human cells where only type I IFN production is inhibited by viral proteins (68). Given the variety of IFN subtypes and bat species, it will be important to further elucidate the response within different immune and structural cell types from multiple bat species following infection with viruses representing diverse viral families.

### Interferon Signaling

In human cells, IFNs interact with IFN α/β receptors (IFNAR), which comprise IFNAR1 and IFNAR2, to induce ISGs. IFN signaling in *P. alecto* cells is dependent on IFNAR2 (69): depletion abolishes IFN signaling and significantly increases replication of H1N1 influenza virus. It is widely accepted that ISG expression correlates with the establishment of an antiviral state in infected and neighboring cells (36). In human cells, based on cell type and duration of IFN treatment, 50-1000 ISGs have been identified (36). It has not yet been established how many ISGs are induced in different bat cells. Furthermore, Shaw and colleagues demonstrated that each mammal possesses a unique repertoire of ISGs, including genes that are common and others that are species or lineage specific (70). Considering that bats are over 1300 species and are distributed between two sub-orders, it is likely that they express unique and different ISGs. Identifying and studying homologs of human ISGs alone may not represent the full potential of ISGs in bats.

Similar to what is seen in human cells, poly(I:C) induces the expression of transcripts for *MDA5, RIG-I*, radical S-adenosyl methionine domain-containing 2 (*RSAD2*), *IRF7*, 2′-5′-oligoadenylate synthase 1 (*OAS1*), IFN-inducible protein 6 (*IFI6*) and myxovirus resistance 1 (*Mx1*) in *E.fuscus* kidney cells (49). *Mx1, OAS1* and protein kinase R (*PKR*) transcritps are also inducible in *P. alecto* bat cells in an IFN dose-dependent manner (71). Furthermore, the *OAS1* gene promoter in *P. alecto* cells has two IFN-stimulated response elements (ISREs), compared to the one ISRE element that is seen in the human OAS1 promoter (71). Thus, OAS1 may play an important antiviral role in RNA virus infections in *P. alecto* and *E. fuscus*. A separate study showed that ectopic expression of Mx1 from six different bat species reduced ebolavirus and influenza A virus replication in human embryonic kidney (HEK293T) cells (72). Residues within the Mx1 protein in 13 species of bats are positively evolving (72), hinting at the importance of their role in limiting virus propagation.

ISG transcript expression kinetics have been studied in *P. alecto* cells. There is a universal rapid induction and subsequent rapid decline in the levels of all ISG transcripts that were studied in type I IFNα treated *P. alecto* cells (73), which may correlate with quicker control of virus replication and reduced cellular toxicity. In contrast, ISG transcript levels in IFN-treated human cells remained elevated for longer times (73). Transcripts for ISGs in unstimulated bat cells were also higher than in human cells, further corroborating the observation of high levels of basal IFNα in this bat species (15).

Unlike in human cells, ribonuclease L (RNase L) is inducible by IFNs in *P. alecto* cells. Expression of atypical ISGs has also been observed in immortalized *P. vampyrus* kidney cells infected with Newcastle disease virus (NDV) (74). In addition to upregulating expression of *IFN*β, *RIG-I, ISG15, MDA5*, and *IRF1*, NDV-infected *P. vampyrus* kidney cells also upregulated transcripts for *RND1*, SERTA-domain containing 1(*SERTAD1*), ChaC glutathione specific gamma-glutamylcyclotransferase 1(*CHAC1*), and *MORC3*. The activation of these genes is dependent on NDV infection, as universal IFNα treatment alone is not sufficient (74). Thus, virus infection may induce secondary responses that augment IFN treatment. Furthermore, bats may have evolved unique sensors of viral components or PAMPs that can activate ISGs in the absence of IFN stimulation. Alternatively, bats may have evolved virus sensing and signaling mechanisms that can stimulate the production of ISGs via transcription factors that directly interact with the promoters of ISGs, independent of IFN production. The expression of RND1 as an ISG in the above study was restricted to *P. vampyrus* cells, while MORC3 expression varied between cells from different bat species, further highlighting the importance of species-specific differences in bats (74). The atypical ISGs that were reported in this study have not been characterized as antiviral genes in bats or other mammals, including humans and murine species. These observations also raise additional questions about the possibility of virus-specific antiviral genes that are induced independently of IFNs in bat cells.

In summary, bats respond to RNA virus infections by inducing a robust IFN response while controlling an exaggerated pro-inflammatory response, thus limiting virus-induced immunopathology as observed in humans infected with these viruses (51, 52, 75). However, these observations have largely been made in primary and immortalized cell lines and the physiological relevance of these responses in *in vivo* bat model systems remains to be tested. Several unique ISGs and atypical induction of ISGs have been observed in bat cells (73). In human cells, expression of individual ISGs can inhibit virus replication, such as ISG20 that displays remarkable anti-bunyavirus activity (76). The effects of expressing homologs of atypical bat ISGs in human cells have not been studied. Learning from atypical antiviral immune responses in bat cells may enable researchers to design alternate therapeutic strategies to induce or exogenously activate antiviral pathways in humans and agricultural animals that are infected with emerging high-impact viruses. Studies in bat cells also highlight the antiviral roles of less studied IFNs, such as IFNκ and IFNω (65). Studies looking at the effect of inducing these IFNs in human cells may help us identify the role played by these IFNs in limiting replication of zoonotic bat-borne viruses. Although responses to RNA virus infections are being investigated in bats, the immunological responses to DNA viruses are less studied.

### Sensing DNA Viruses

The focus on zoonotic RNA viruses has led to slower developments in understanding interactions between the bat immune system and DNA viruses. Multiple DNA viruses have been detected in bats, including herpesviruses (77–80), adenoviruses (81), hepadnaviruses (82), poxviruses (83) and polyomaviruses (81, 84, 85). These discoveries highlight the need to study antiviral responses to DNA viruses in bats. In human cells, endosomal TLR9 (35) and cytosolic receptors from the PYHIN family (86) [absent in melanoma 2 (AIM2), IFN-inducible gene 16 (IFI16), myeloid cell nuclear differentiation antigen (MNDA) and IFN-inducible protein X (IFIX)], cyclic GMP-AMP synthase (cGAS) (87), DNA-dependent activator of IFN-regulatory factor (DAI) (88), RNA polymerase III (PolIII) (89), LRR binding FLII interacting protein 1 (Lrrfip1) (90), DDX41 (91), DExH-Box helicase 9 (DHX9) and DEAH-Box helicase 36 (DHX36) (92) can detect exogenous or self DNA (86, 93). Detection of exogenous or self DNA leads to activation of downstream mediators and expression of antiviral and pro-inflammatory cytokines (35).

### Dampened DNA Virus Sensing in Bats

**Figure 2:**
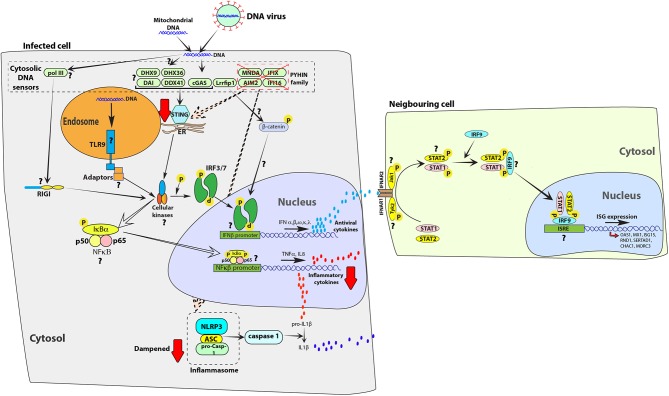
Exogenous and self-DNA sensing pathways are dampened in bat cells. In human cells, endosomal TLR9 (35) and cytosolic receptors from the PYHIN family (86) [absent in melanoma 2 (AIM2), IFN-inducible gene 16 (IFI16), myeloid cell nuclear differentiation antigen (MNDA) and IFN-inducible protein X (IFIX)], cyclic GMP-AMP synthase (cGAS) (87), DNA-dependent activator of IFN-regulatory factor (DAI) (88), RNA polymerase III (PolIII) (89), LRR binding FLII interacting protein 1 (Lrrfip1) (90), DDX41 (91), DExH-Box helicase 9 (DHX9) and DEAH-Box helicase 36 (DHX36) (92) can detect exogenous and self-DNA (86, 93). On binding to DNA, these receptors signal through adaptor proteins to activate cellular kinases, such as TANK-binding kinase 1 protein (TBK1), which in-turn activates transcription factors, such as interferon regulatory factor 3 (IRF3) or IRF7 and nuclear factor kappa-light-chain-enhancer of activated B cells (NFκB) to induce expression of antiviral interferons (IFNs) and pro-inflammatory cytokines, respectively. PYHIN family of cytosolic receptors signal through STING and the NLRP3 inflammasome. This family of receptors has been negatively selected for and lost in the genomic sequences of bats (94). A downstream signal mediator of cGAS, stimulator of IFN genes (STING) is less functional in bat cells, relative to human cells (95). The attenuated function of STING would likely extend to other DNA sensors that signal through STING, such as DDX41, DHX9, DHX36, and DAI. The presence and function of additional homologs of DNA sensors, such as TLR9, PolIII, and Lrrfip1 have not been characterized in bats. In the figure, red arrows indicate a dampened response in the pathway, relative to human cells. Question marks (**?**) highlight pathways and molecular homologs that have not yet been characterized or identified in bats. The data have been compiled from studies in different species and one finding may not represent a universal bat response. ER, endoplasmic reticulum.

While bats have evolved to detect and respond to RNA virus infections, studies indicate that responses to DNA viruses are dampened (94, 95) (Figure 2). Ahn et al. compared the genomic sequences of 10 bat species and discovered that the PYHIN family of genes were absent (94). In addition, a recent study found that the ability of stimulator of IFN genes (STING)—which is an essential adaptor protein that is involved in multiple DNA sensing pathways (93)—to induce IFN expression is dampened in bat cells due to the loss of a serine residue at position 358 (95). This mutation in STING led to higher levels of herpes simplex virus (HSV) replication in *P. alecto* kidney cells and re-introducing the serine residue at position 358 significantly inhibited HSV replication. These studies provide experimental support for the loss of self DNA and exogenous DNA sensing and signaling in bat cells.

TLR9 sequences in eight bats, from three different families (*Pteropodidae, Vespertilionidae*, and *Phyllostomidae*) are evolving under purifying selection and multiple mutations in the ligand-binding domain of this receptor have been reported (96). TLR9 in *E. fuscus* kidney cells could not be stimulated to the same extent as observed in human cells by CpG ODNs (49). This observation supports computational data by Escalera-Zamudio et al. that TLR9 in bats may have evolved altered ligand specificity (96) and bat-specific CpG ODNs may be required to initiate TLR9 signaling. The functional roles and activating ligands of all TLRs in bats are yet to be identified.

The reduced ability of bat cells to detect exogenous and self-DNA is speculated to be a side-effect of the evolution of flight (94). During flight, the body temperature of bats can rise dramatically to over 41°C (97). High metabolic rates, along with elevated body temperatures, produce reactive oxygen species, which can cause DNA damage and release of DNA into the cytoplasm (98, 99). To overcome this, bats show evidence of positive selection in various genes involved with DNA repair, with consequences for antiviral responses (29). Similarly, the selective pressure of DNA damage and release of self-DNA in the cytoplasm may have selected for the loss of certain cytosolic sensors of DNA in bat cells, while evolving DNA binding motifs in other receptors, such as TLR9 (96). These observations have been largely made by analyzing genomic sequences and they raise several questions about antiviral defensive responses in bats against DNA viruses. Receptors such as Lrrfip1 (90), TLR9 (96) and polIII (89) may have been positively selected for in bats to compensate for the loss of the PYHIN family of proteins and the lack of activation of STING (Figure 2). In addition, other pathways, such as autophagy may have undergone positive selection in bats (100). The DNA-dependent protein kinase catalytic subunit (DNA-PKcs, encoded by *PRKDC*) is involved in DNA-damage responses and has additionally been suggested to bind cytosolic DNA and promote type I IFN responses (101). DNA-PKcs is positively selected for in the genomes of both *P. alecto* and *M. davidii*, likely due to the evolution of flight (29). Whether positive selection on bat DNA-PKcs has inadvertent consequences for DNA sensing in bats remains to be determined.

### Compensating for Dampened DNA Sensing

There is evidence of crosstalk between viral RNA- and DNA-sensing pathways in mammals and there also appears to be positive feedback mechanisms that increase cellular expression of STING (102). In theory, mRNA transcripts from DNA viruses could be sensed by RIG-I in bat cells to initiate an antiviral response, which could lead to increased expression of STING, thereby overcoming the limited activation of STING that is typically seen in bat cells. It is unlikely that bats have lost all forms of exogenous and endogenous DNA sensing machinery since DNA viruses have been detected and isolated from multiple species of bats (77–79, 83, 103). Future studies will elucidate adaptations that bat cells have evolved to sense DNA viruses, while limiting detection of self DNA.

Nucleotide-binding oligomerization domain (NOD)-like receptors are intracellular PRRs that can recognize nucleic acids from invading viruses (104). Genes encoding the NOD-like receptors NACHT, LRR, and PYD domains-containing protein 3 (NLRP3) and NOD-, LRR- and CARD-containing 5 (NLRC5) have been identified in the transcriptome of *P. alecto* (37) but only NLRP3 has been functionally characterized in bats (17). Despite the identification of homologs of several PRRs in some bat species, the available genomes of most bat species have not been assessed for the presence of PRRs. The presence or absence of novel immune-related genes or altered functions for existing genes have not been exhaustively studied. Similarly, it is unknown if existing PRRs can recognize a broader category of PAMPs to compensate for the lost PRRs in bats.

In summary, the evolution of flight in bats may have had inadvertent consequences for their immune responses. Bats diverged over 80 million years ago (21, 105) and are the only mammals capable of true self-powered flight (105). Bats display high metabolic rates and body temperatures during flight (106). To minimize DNA damage that is associated with high metabolic rates, DNA repair pathways have been positively selected for in bats (29). In addition, endogenous DNA sensing pathways have been dampened to reduce self-DNA-mediated immunopathology (94, 95). This may have had consequences for detecting viral DNA. Whether global DNA sensing pathways in bats have been dampened is speculative, especially since bats carry several DNA viruses and do not display overt signs of infection (43). Future studies will determine if bats have evolved novel mechanisms to differentially sense and respond to self and exogenous DNA.

## Adaptive Immunity in Bats

There are limited studies on adaptive immune responses in bats, largely due to a lack of reagents and appropriate experimental models. Recent developments in the identification and development of bat cross-reactive antibodies along with the establishment of various captive bat experimental colonies have facilitated advancement in this area, as we discuss below.

### Antibody Responses in Bats

Transcripts for major subclasses of antibodies, such as IgA, IgE, IgG, and IgM have been detected in bats (107, 108). Serological studies of virus-specific bat antibodies were among the first functional studies to be performed. Infection of *R. aegyptiacus* with MARV resulted in the development of antigen-specific IgG responses in the bats by 28 days after challenge (25, 109–111). While the durability of the antibody response seems to vary—with one study reporting that antibody titers fell below detectable levels by 3 months after infection (25) and another study finding virus-specific IgG levels were still maintained 11 months following infection (111)—both studies found that secondary challenge with MARV increased virus-specific IgG antibodies titres to a greater extent than seen following initial MARV infection. Earlier studies reported that the magnitude and duration of antibody responses in bats in response to antigens such as X174 bacteriophage or sheep red blood cells may be lower compared to what is seen in conventional laboratory animals (112, 113). The function of antibodies during viral infection in bats is also not known. While seronegative bats had no detectable virus replication or shedding (25), a subsequent study identified non-neutralizing antibody responses following MARV, EBOV, and Sosuga virus (SOSV) infection (114). In another study, sera from wild-caught *E. fuscus* bats that were positive for *E. fuscus* gammaherpesvirus (EfHV) genomic sequences did not contain neutralizing antibodies against the virus (79). Moreover, experimental infection of *P. alecto* with Hendra virus led to inconsistent patterns in seroconversion (28). Approximately half of the animals seroconverted and had relatively low titres of virus-neutralizing antibody, but the bats were not necessarily protected from virus replication and shedding. These data suggest that the antibodies that arise in bats in response to virus infection may control viruses via a mechanism that is independent of virus neutralization.

Although patterns of seroconversion in bats have been inconsistent at the individual-level, serological data may provide valuable information about virus circulation at the population level. For example, age-specific seroprevalence against Hendra virus in *P. scapulatus* populations followed the classic J-shaped curve suggesting waning maternal immunity followed by horizontal transmission (115). Currently, our understanding of the within-host dynamics of viruses within bats is poor—a spectrum of hypotheses from acute infections followed by long-term immunity to persistent infections followed by latency and reactivation are possible (116). Serological data can help distinguish among possible hypotheses to identify mechanisms of viral persistence and circulation in bats. For example, Glennon et al. constructed a generalized SEIR (susceptible, exposed, infectious, recovered) model and demonstrated 46 plausible transitions among SEIR states in infected bat populations. When they fitted all 46 models to longitudinal data on henipavirus serology from captive *Eidolon helvum* bats in Ghana, transitions that involved re-infections and latent infections best fit the data (117). Similarly, Brook et al. fitted serological data of bat species in Madagascar to mathematical models and found support for waning maternal immunity in neonates, whereas in adult bats the models supported the continued presence of immunity (118). More recently, however, *R. aegyptiacus* challenged with MARV 17-24 months after primary exposure exhibited a robust MARV-specific antibody response and no detectable viremia or oral shedding of virus even though the bats had MARV-specific antibodies below the threshold of seropositivity (25). Following heterologous MARV challenge, bats previously exposed to MARV exhibited some virus replication, but no detectable virus shedding. This finding suggests that although antibody levels may wane over time, bats still maintain protective immunity.

At the genomic level, bats appear to have a much larger repertoire of germline genes encoding immunoglobulin variable (V), diversity (D) and joining (J) segments than humans, which could potentially provide a larger number of antigen specificities in their naive B cell receptor (BCR) repertoire. In little brown bats (*Myotis lucifugus*), there is less evidence of somatic hypermutation, indicating that bats may rely more on their germline repertoire to respond to infections (107, 119).

### Immune Cell Populations in Bats

Very few bat-specific antibodies that identify immune cell populations exist. To overcome this obstacle, Martinez Gomez et al. and Periasamy et al. screened commercially available antibodies for cross-reactivity to cells isolated from blood and primary and secondary lymphoid tissues of *P. alecto* bats (120, 121). Using monoclonal antibodies specific to mammalian transcription factors, Martinez Gomez et al. found that wild-caught *P. alecto* bats displayed a predominance of CD8+ T cells in the spleen, whereas CD4+ T cells were the most prevalent lymphocyte in the blood, lymph nodes, and bone marrow. An unexpectedly high portion of CD3^+^ T cells constitutively expressed mRNAs for IL-17A, IL-22 or transforming growth factor beta 1 (TGFβ1), which indicates a strong bias toward Th17 and regulatory T cell subsets in bats. Following stimulation with mitogens—phorbol 12, 13-dibutyrate (PDBu) and ionomycin, the total number of CD3^+^ T cells expressing IL-17A, IL-22, or TGFβ1 did not increase, but there was an increase in the frequency of T cells expressing TNF, IL-10, IFNγ, IL-2, granzyme B, and perforin.

To evaluate B cell responses in bats, antibodies that recognize bat Ig, MHC-II, CD21, and CD27 were identified (121). B cells were successfully identified, although it was found that unlike humans, bats have more T cells than B cells in the blood and spleens. B cell proliferation was induced by lipopolysaccharide (LPS) treatment in bats suggesting they may have a functional TLR4 homolog. Furthermore, calcium influx was observed upon crosslinking of the BCR, suggesting that bat B cells are functional. The experiments performed on *P. alecto* were done on wild-caught bats, which may differ in their immune status and this is evident by the variability in data observed in this study within sampled bats (120); however, Periasamy *et al*. also evaluated captive *Eonycteris spelaea* bats and again found that T cells are the dominant immune cell population in the spleen and blood (121).

**Figure 3:**
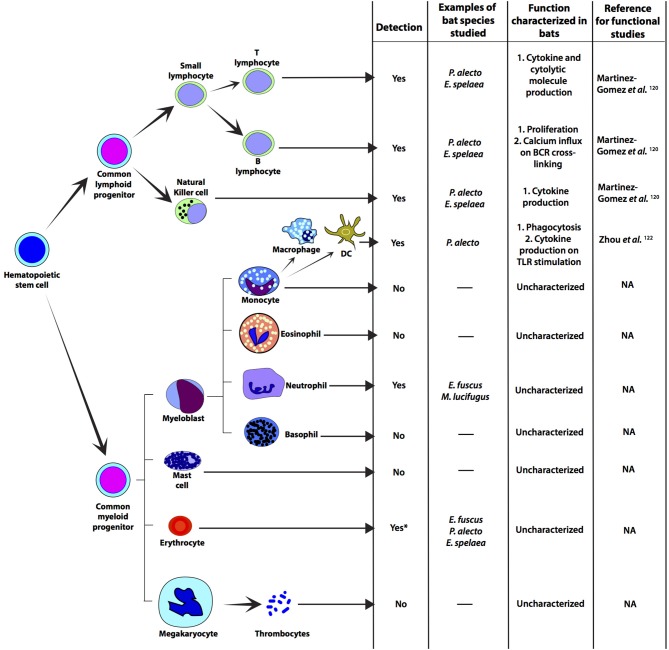
Immune relevant cells that have been detected in representative bat species. Progenitor cells and cellular differentiation have not been studied in bats, but there are some studies on myeloid and lymphoid cells. Most studies on bat immune-relevant cells have been carried out on cells from fruit bats. Functional characterization of bat T and B cells have been carried out by detecting cytokines that are secreted by these cells on stimulation (120, 121). NK cells from *P. alecto* and *E. spalaea* have been detected by flow-cytometry (121). Macrophages from *P. alecto* have been detected and cultured *in vitro* by using *P. alecto*-specific reagents. Functional characterization of *P. alecto* macrophages have been carried out by stimulating them with TLR3 ligand [poly(I:C)] and TLR7/8 ligand (CL097) (122). Neutrophils have been detected in *E. fuscus* and *M. lucifugus* by differential staining (49, 123). Thrombocytes have not been characterized, but the ability of bats to heal their wounds has been studied (124). *Depletion of red blood cells (erythrocytes; RBCs) from bat samples have been reported, but RBCs from bats have not been functionally characterized. NA, not applicable; DC, monocyte derived dendritic cell; TLR, Toll-like receptor.

In the absence of reliable *in vivo* studies, attempts have been made to decipher adaptive immunity in bats using primary cells, immortalized cell lines and *ex vivo* cell cultures (Figure 3). Crude bone-marrow derived myeloid cell preparations from *E. fuscus* were used to validated dsRNA sensing (49). *P. alecto* kidney cells were used to identify self and Hendra virus peptide presentation by MHC class I molecules (125). This study showed that bat MHC molecules can accommodate larger peptides, compared to other mammals and have unique consensus-binding motifs, potentially as a result of their co-evolution with viruses (125). The MHC class I protein complexes (126) in *P. alecto* were recently crystallized, revealing three additional amino acids in bat MHC class I (methionine, aspartic acid and leucine), compared to other selected mammals (127). The three amino acids formed an additional salt bridge that could potentially present high affinity peptides during the peptide exchange process in bat cells facilitating an efficient cell-mediated immune response. With the ongoing development of bat-specific reagents, bone marrow derived dendritic cells (DCs) and macrophages have been cultured *in vitro* from *P. alecto* (122). Zhou et al. generated *P. alecto-*specific reagents, such as granulocyte-macrophage colony-stimulating factor (GM-CSF), interleukin 4 (IL-4), FMS-like tyrosine kinase 3 ligand (FLT3L) and colony-stimulating factor 1 (CSF-1) to culture and characterize monocyte-derived DCs, conventional DCs (cDCs) and macrophages from *P. alecto* (122). Zhou et al. demonstrated that similar to human and rodent cells, bat macrophages, putative monocytes and putative cDCs are phagocytic. On stimulation with poly(I:C) (TLR3 ligand), Mx1 (ISG) transcript levels increased in all three cell types; however, FLT3L-generated bat bone-marrow-derived bat DCs induced high levels of IFNλ2 transcripts on poly(I:C) stimulation. This is consistent with observations in mice and humans suggesting that bone-marrow-derived bat DCs share this functional specialization with rodents and humans (122).

Although reagents are now being identified and developed that can be used to study bat immunology, experiments evaluating the immune response to infection still require specialized facilities to house captive or wild-caught bats. To overcome this challenge, 80-100 chimeric bat-mice were developed to study the bat immune system by reconstituting bat immune cells in these mice. Splenocytes and bone marrow cells from *E. spelaea* were transplanted in immunodeficient mice (NOD-scid IL-2R -/; NOD scid gamma mouse, NSG) (128). Bat immune cells successfully repopulated the bone marrow, spleen, liver and blood without the development of graft rejection. Furthermore, chimeric bat-mice were able to respond to immune stimuli and produce antigen-specific antibody responses. Of note, although these mice were successfully reconstituted with bat immune cells, the proportion of immune cell subsets does not recapitulate the immune cell frequencies observed *in vivo* in bats thus far. Even with that caveat, the ability to create 80–100 mice with reconstituted bat immune cells (128) provides an excellent foundation to better study immune cell responses to infection in bats.

## Modulation of Antiviral Responses

Viruses evolve mechanisms to counteract cellular antiviral responses in the host (129, 130). Emerging bat-borne viruses, such as coronaviruses that cause SARS and MERS inhibit innate antiviral responses in the infected host while inducing a strong pro-inflammatory cytokine response that is associated with immunopathology and significant morbidity and mortality (52, 131, 132). The ability of bats to harbor viruses from many viral families with no overt signs of disease may hint at the inability of these viruses to modulate antiviral responses in bats. Although *in vivo* studies indicate that bats infected with henipaviruses do not develop clinical signs of disease (27, 28), henipavirus infection of *P. alecto* lung cells inhibits IFN production and signaling. Ectopic expression of the ISG tetherin from fruit bats (*Hypsignathus monstrosus* and *Epomops buettikoferi*) can inhibit Nipah virus replication in fruit bat cells (133). However, experimental Nipah virus infection of bat cells has been shown to inhibit IFN production (68) and possibly, the associated downstream expression of ISGs, such as tetherin. Thus, further investigations are required to identify how bats control infection with henipaviruses at cellular and systemic levels in the potential absence of IFNs.

Not all bat-borne viruses can inhibit IFN production and signaling in bat cells. MERS-CoV is speculated to have evolved in Vespertilionid bats (12). MERS-CoV proteins can inhibit IFN production in human cells (134–136), which contributes to its pathology. However, infection of *E. fuscus* kidney cells with MERS-CoV induces the expression of IFNβ and OAS1 transcripts in an IRF3-dependent manner (59). Infection of Jamaican fruit bats (*A. jamaicensis*) with MERS-CoV did not produce apparent disease symptoms and induced the expression of ISGs, such as Mx1, ISG56 and CCL5 (26), further bolstering the *in vitro* observations seen with *E. fuscus* kidney cells. It is likely that studies with a human adapted isolate of MERS-CoV (EMC/2012) do not represent true virus-bat interactions. Future studies with bat CoV isolates will shed more light on the ability of bat CoVs to modulate antiviral responses in bat cells. The ability of bat cells to resist virus protein-mediated modulation of innate defensive responses is the focus of ongoing studies. The 3ABC proteases of human hepatitis viruses cleave MAVS to evade the innate immune response (137). A recent study demonstrated that bat MAVS orthologs are relatively resistant to cleavage by their cognate 3ABC proteases, whereas proteases from bat-borne viruses retain the ability to cleave human MAVS (62).

There are limited studies that have studied the functional conservation and ability of bat cellular molecules to stimulate antiviral signaling pathways in human cells. The above study by Feng *et al*. showed that in addition to being resistant to cleavage by 3ABC proteases, bat MAVS can actively signal in human cells to activate the IFN promoter (62). The idea of using bat molecules that are resistant to viral protein-mediated modulation as therapeutics is still far-fetched. Further studies are required to assess the functional conservation of other bat molecules that may activate endogenous innate immune responses in spillover mammals, such as humans that are infected with bat-borne viruses.

As a reservoir host of several viruses, bats have evolved to counteract the immune modulatory effects of viral proteins. Limited studies have been carried out to understand how innate and intrinsic antiviral responses in bat cells circumvent inactivation by viral proteins. The few studies that have attempted to answer this question have used human virus isolates that are likely adapted to infect and modulate antiviral responses in human cells. Studies with these isolates [MERS-CoV/ EMC2012 (26, 59), Nipah virus/ Bangladesh/ human/ 2004/ Rajbari R1 (68) and Nipah virus/ Malaysia/ human/ 1000/ PKL (68)] do not represent true virus-host interactions that are occurring in the natural setting. Given the limitations and challenges associated with isolating viruses from bats (138), these studies represent our best understanding of these interactions. Isolating bat viruses, such as recently discovered bat ebolaviruses (6, 7, 139) and propagating them in relevant bat cells will represent true virus-host interactions and selection pressure.

## Immune Response and Virus Spillover

Understanding host immune responses in bats and developing effective tools to detect and measure these responses in a meaningful way at a population level will allow for the study of variations in antiviral responses in wild bat populations. Wild bat populations are vulnerable to multiple environmental stressors, including seasonal fluctuations in food availability, climatic-stressors, and anthropogenic disturbances (140). Each of these environmental stressors can affect multiple barriers to pathogen spillover (23, 141). For example, viral excretion dynamics may be driven by bat densities and contact with humans may be influenced by bat distributions (142). The effect of environmental stressors on immune defense and subsequent pathogen shedding is an important phenomenon for spillover. Pulses of Hendra virus excretion from Pteropodid bats, and associated Hendra virus spillover to horses, have been coincident with severe food shortages for bats that are driven by climatic anomalies (143). Moreover, seasonal life-history events such as pregnancy and lactation can induce physiological and energetic stress that influences antiviral activity (144). Connections among nutritional stress, reproductive stress and increased Hendra virus seroprevalence were observed in little red flying foxes, *Pteropus scapulatus* (115). Elucidating the links among nutritional and physiological stress, and mechanisms underlying these associations require extensive temporal and spatial sampling of wild bat populations in concert with laboratory and modeling studies.

A large proportion of *E. fuscus* bats are infected with an *E. fuscus* gammaherpesvirus (79) and it is unknown if the lack of an effective antiviral response against DNA viruses aids in inter- and intra-species transmission of herpesviruses within bats. Speculations remain about the bat antiviral response and the advantages of co-existing with some of these viruses. Could infection with these viruses prime bat antiviral responses against pathogens that do kill bats, such as Tacaribe virus and rabies virus (145, 146)? Experimental rabies virus infection studies in bats have led to conflicting conclusions about the nature of disease, ranging from asymptomatic infections to fatal meningitis (146–148). Perhaps different strains of rabies virus, the infectious dose and the route of exposure have varying effects on the outcome of infection in different bat species. Currently, the literature is biased toward studying viruses that do not cause visible signs of disease in bats. Indeed, there is a need to study viruses that cause disease in bats and associated immunological consequences in these bats. Bats can remain infected with multiple zoonotic viruses in the wild (22), the role of co-infection in modulating immune responses in bats needs to be investigated. Understanding the impact of deforestation, nutrition and other environmental factors on antiviral responses in bats and correlation with virus spillover may allow for the development of conservation policies that will mitigate the risk of virus spillover from bats into humans and agricultural animals.

## Challenges in Studying Bats

As mentioned previously, the order Chiroptera is diverse and consists of over 1300 species of bats (18). The two suborders, Yinpterochiroptera (which comprises megabats and several families of microbats) and Yangochiroptera (comprising all remaining microbat families) diverged over 50 million years ago (19–21) and within these suborders, bat species display tremendous diversity in size, morphology, ecological niches, diets, and social interactions. The large species diversity is also represented, in parts, in the evolution of immune responses in bats. For example, although primary cells from *P. alecto* constitutively express IFNα (15), Pavlovich et al. did not detect constitutive expression of IFNα in cells from *R. aegyptiacus* (16). Furthermore, Pavlovich et al. identified an expansion of type I IFN genes in *R. aegyptiacus* (16), whereas type I IFN locus is contracted in *P. alecto* (15).

The limitations of studying bat-virus interactions also extend to the tools that are currently available. The limited repertoire of cell lines from few species of bats and their tissues have led to studies where viruses have been propagated and studied in cell lines that are derived from unrelated or closely related species of bats. This important topic was recently reviewed by us and a call for collaboration and sharing of resources was put out to advance bat-virus interaction studies at a faster pace (138). Similarly, inability to isolate majority of the “detected” bat viruses have led to studies in bats and bat cells with human isolates of closely related viruses (24, 26). These studies have demonstrated that infected bats do not develop disease, raising speculations on the role of bats as reservoirs. However, these observations have been made in limited species of bats. Isolating a bat virus and investigating virus replication and pathogen-host interactions in cells from the source species of bat will represent true natural processes and enable us to better predict factors that lead to pulses of increased virus replication and shedding in bats (116, 149). An interesting study to investigate species specific antiviral responses would be to infect multiple bat species with Tacaribe virus and monitor the outcome of infection. Tacaribe virus causes a fatal infection in Artibeus bats (145) and it will be interesting to see if this outcome can be reproduced in other species of bats.

Most infection studies in bats and bat cells have used human isolates or virus stocks that have been propagated in non-bat cell lines (138). Propagating viruses in non-natural hosts generates adaptive mutations, such as mutations that were detected during sequential passages of Marburg virus in mice and cell-culture (150). Thus, over time, lab cultures of viruses do not represent viruses that were originally detected or isolated in bats. However, even with these limitations, they are currently the best model strains that we have. Indeed, there is a need to isolate bat viruses and generate virus stocks that have been propagated in cells from the same species of bat. This was recently achieved for a bat gammaherpesvirus that was isolated from *E. fuscus* using a kidney cell line that was generated from the same species of bat (79).

It has been particularly challenging to isolate bat viruses and only fragments of viral genomes have been detected for multiple bat-borne viruses (6, 7, 85, 151). The use of reverse genetics and molecular tools have allowed researchers to rescue bat influenza viruses (152). A similar approach, if possible, could be developed for other bat viruses that have been difficult to isolate. This will also require developing cell lines and reagents for the same species of bat from which virus isolation is to be attempted.

In summary, studies in bats and bat cells are challenging since limited tools are currently available. Lack of susceptible cell lines have led to maintenance and propagation of bat viruses in cells from other mammalian species, which could, in theory, lead to adaptive mutations in bat viruses. However, even with these caveats, the field of bat immunology and virology is slowly developing and discovering novel adaptations in the bat immune system that is redefining our understanding of mammalian immune responses (17, 94, 95, 100).

## Conclusion

Bats have emerged as important reservoirs of zoonotic viruses that cause serious disease in humans and other animals with no visible clinical signs of disease in bats (153). This has prompted scientists to study the evolution of antiviral responses in bats to understand their ability to tolerate infections with viruses that are lethal in other mammals. Several adaptations have been discovered in bat cells that enable robust antiviral immune responses against RNA viruses. However, the immune response against DNA viruses in bats has been dampened (17, 94), most likely due to the evolution of flight in bats and the associated risk of DNA damage driving immunopathology. Further studies are required to validate the physiological and systemic outcomes of a “dampened” antiviral response to DNA viruses in a bat *in vivo* model. In addition, alternative mechanisms of DNA virus sensing and the enhancement of TLR9 signaling in bat cells need to be validated by additional studies.

Studies have shown that bats are resistant to MERS-CoV protein-mediated modulation of antiviral responses (26, 59). Similar studies need to be undertaken for other bat-borne viruses. It is important to understand how antiviral responses in bats have evolved to inhibit virus protein-mediated modulation. Insights from these studies will inform strategies to identify novel drug targets in spillover species, including humans.

As more bat reagents become available in the future, it should be possible to elucidate the mechanisms that bats have evolved to coexist with viruses in the absence of disease and this information could be useful for preventing or treating lethal viral infections in humans (55, 154).

## Author Contributions

AB, MB, KK, and KM wrote the first draft. All authors edited and contributed to subsequent drafts.

### Conflict of Interest

The authors declare that the research was conducted in the absence of any commercial or financial relationships that could be construed as a potential conflict of interest.
